# Redetermination of loperamide monohydrate

**DOI:** 10.1107/S1600536812003091

**Published:** 2012-01-31

**Authors:** Jerry P. Jasinski, Curtis J. Guild, A. S. Dayananda, H. S. Yathirajan, A. R. Ramesha

**Affiliations:** a Department of Chemistry, Keene State College, 229 Main Street, Keene, NH 03435-2001, USA; b Department of Studies in Chemistry, University of Mysore, Manasagangotri, Mysore 570 006, India; c R. L. Fine Chem, Bengaluru, 560 064, India

## Abstract

The structure of the title compound {systematic name: 4-[4-(4-chloro­phen­yl)-4-hy­droxy­piperidin-1-yl]-*N*,*N*-dimethyl-2,2-di­phenyl­butanamide monohydrate}, C_29_H_33_ClN_2_O_2_·H_2_O, has been redetermined at 170 (2) K. The redetermination is of significantly higher precision than the previous structure determination at room temperature and includes the H-atom coordinates that were not included in the previous report [Germain *et al.* (1977 ▶). *Acta Cryst*. B**33**, 942–944]. It consists of a piperidin-1-yl ring in a distorted chair conformation, with the *N*,*N*-dimethyl-α,α-diphenyl­butyramide and the 4-chloro­phenyl and hy­droxy groups bonded in *para* positions and an external water mol­ecule within the asymmetric unit. The dihedral angles between the mean plane of the piperidine ring and the 4-chloro­phenyl and two benzene rings are 83.4 (5), 76.4 (2) and 85.9 (2)°, respectively. The two benzene rings are inclined to one another by 50.8 (6)°. In the crystal, mol­ecules are linked by O—H⋯O and O—H⋯N hydrogen bonds and weak C—H⋯O intermolecular interactions, forming an infinite two-dimensional network along [110].

## Related literature

For the pharmacological properties and therapeutic efficacy of loperamide, see: Heel *et al.* (1978 ▶). For the crystal structure of loperamide hydro­chloride tetra­hydrate, see: Caira *et al.* (1995 ▶). For the crystal structure of loperamide *N*-oxide hydrate, see: Peeters *et al.* (1996 ▶). For the crystal structure of the title compound, see: Germain *et al.* (1977 ▶). For puckering parameters, see: Cremer & Pople (1975 ▶).
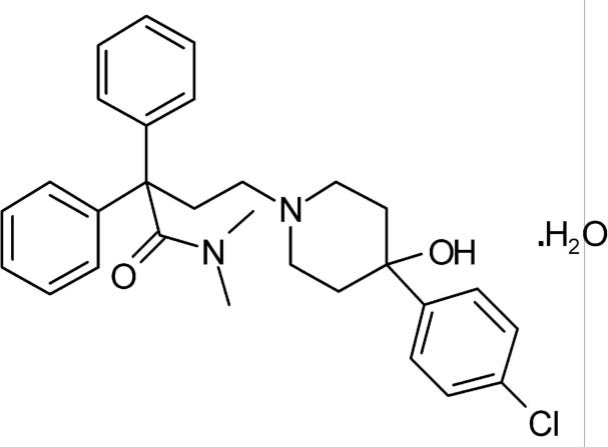


## Experimental

### 

#### Crystal data

C_29_H_33_ClN_2_O_2_·H_2_O
*M* *_r_* = 495.04
Orthorhombic, 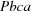

*a* = 16.7869 (4) Å
*b* = 15.1506 (6) Å
*c* = 20.6617 (6) Å
*V* = 5254.9 (3) Å^3^
*Z* = 8
Mo *K*α radiation
μ = 0.18 mm^−1^
*T* = 170 K
0.45 × 0.30 × 0.20 mm

#### Data collection

Oxford Diffraction Xcalibur Eos Gemini diffractometer
Absorption correction: multi-scan (*CrysAlis RED*; Oxford Diffraction, 2010 ▶) *T*_min_ = 0.989, *T*_max_ = 1.000
48373 measured reflections
6247 independent reflections
5231 reflections with *I* > 2σ(*I*)
*R*_int_ = 0.033

#### Refinement

*R*[*F* ^2^ > 2σ(*F* ^2^)] = 0.044
*wR*(*F* ^2^) = 0.107
*S* = 1.04
6247 reflections
330 parameters
4 restraints
H atoms treated by a mixture of independent and constrained refinement
Δρ_max_ = 0.27 e Å^−3^
Δρ_min_ = −0.29 e Å^−3^

### 

Data collection: *CrysAlis PRO* (Oxford Diffraction, 2010 ▶); cell refinement: *CrysAlis PRO*; data reduction: *CrysAlis RED* (Oxford Diffraction, 2010 ▶); program(s) used to solve structure: *SHELXS97* (Sheldrick, 2008 ▶); program(s) used to refine structure: *SHELXL97* (Sheldrick, 2008 ▶); molecular graphics: *SHELXTL* (Sheldrick, 2008 ▶) and *DIAMOND* (Brandenburg, 1998 ▶); software used to prepare material for publication: *SHELXTL*.

## Supplementary Material

Crystal structure: contains datablock(s) global, I. DOI: 10.1107/S1600536812003091/lx2225sup1.cif

Structure factors: contains datablock(s) I. DOI: 10.1107/S1600536812003091/lx2225Isup2.hkl

Supplementary material file. DOI: 10.1107/S1600536812003091/lx2225Isup3.cml

Additional supplementary materials:  crystallographic information; 3D view; checkCIF report

## Figures and Tables

**Table 1:** Hydrogen-bond geometry (Å, °)

*D*—H⋯*A*	*D*—H	H⋯*A*	*D*⋯*A*	*D*—H⋯*A*
O1*W*—H2*W*⋯O2^i^	0.89 (2)	2.10 (2)	2.9684 (19)	168 (3)
O1*W*—H1*W*⋯N2	0.90 (2)	2.06 (2)	2.9132 (18)	160 (2)
O2—H2O⋯O1^ii^	0.83 (2)	1.97 (2)	2.7333 (15)	153 (2)
C28—H28*B*⋯O1*W*^iii^	0.96	2.42	3.369 (3)	171

Symmetry codes: (i) 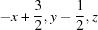; (ii) 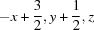; (iii) 
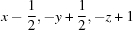
.

**Fig. 1.**
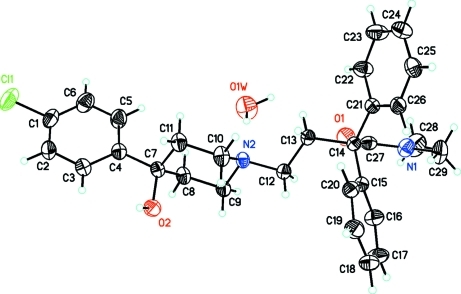
Molecular structure of the title compound showing the atom labeling scheme and 50% probability displacement ellipsoids. H atoms are presented as small spheres of arbitrary radius.

**Fig. 2.**
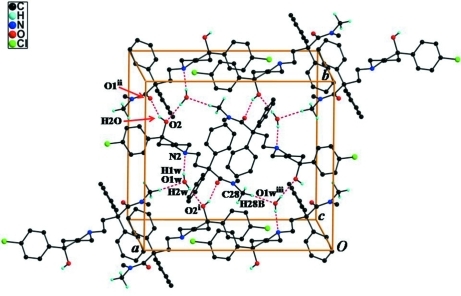
A view of the Ow—H···O, Ow—H···N, O—H···O and C—H···Ow interactions (dotted lines, Ow : H2O) in the crystal structure of the title compound. H atoms non–participating in hydrogen–bonding were omitted for clarity. [Symmetry codes : (i) -x + 3/2, y - 1/2, z; (ii) - x + 3/2, y + 1/2, z; (iii) x - 1/2, - y + 1/2, - z + 1.]

## supplementary crystallographic information

### Comment

Loperamide, a butyramide derivative is a new agent for use in symptomatic control of acute non-specific diarrhoea and chronic diarrhoea. Loperamide is a synthetic piperidine derivative, an opioid drug effective against diarrhea resulting from gastroenteritis or inflammatory bowel disease. Loperamide is an opioid-receptor agonist and acts on the µ-opioid receptors in the myenteric plexus of the large intestine; by itself it does not affect the central nervous system like other opioids. A review of its pharmacological properties and therapeutic efficacy in diarrhoea is reported (Heel *et al.*, 1978). The crystal structures of loperamide hydrochloride tetrahydrate (Caira *et al.*, 1995) and loperamide N-oxide hydrate (Peeters *et al.*, 1996) have been reported. The crystal structure of the title compound was first reported [Germain, *et al.* (1977) *Acta Cryst.* B33, 942–944] with an R value of 9% at room temperature. The present paper is a redetermination of the title compound, C_29_H_33_ClN_2_O_2_Cl . H_2_O, at 170 (2) K with a high accuracy, hydrogen atom coordinates and an R value of 4.39%.

The title compound, is a synthetic piperidine derivative, an opioid drug effective against diarrhea resulting from gastroenteritis or inflammatory bowel disease. It consists of a piperidin–1–yl ring (distorted chair conformation with (Cremer & Pople, 1975) puckering parameters Q, θ and φ of 0.5771 (4)Å, 173.87 (14)° and 195.2 (14)°), with N,N-dimethyl-α,α-diphenylbutyramide and p-chlorophenyl and hydroxy groups bonded in para positions and an external water molecule within the asymmetric unit (Fig. 1). The dihedral angles between the mean planes of the piperidin–1–yl ring, chlorophenyl and two benzene rings are 83.4 (5)°, 76.4 (2)° and 85.9 (2)°, respectively. The two benzene rings are separated by 50.8 (6)°. Crystal packing (fig. 2) is stabilized by O2—H2O···O1 hydrogen bonds from the molecule along with O1w—H2w···O2 and O1w—H1w···N2 hydrogen bonds and weak C28—H28B···O1w intermolecular interactions between the monohydrate water molecule and the title compound (Table 1) forming an infinite 2–D network along [110].

### Experimental

The title compound was obtained as a gift sample from R. L. Fine Chem, Bangalore. X–ray quality crystals were obtained by slow evaporation of dimethylformamide solution (m.p.: 403-407 K).

### Refinement

H1w, H2w and H2O were located by a difference map and refined isotropically. Ow1 with H1w and H2w were set to DFIX = 0.85 (2)Å, while Hw1 and Hw2 were set to 1.35 (2)Å. H20 with O2 was set to 0.82 (2)Å. All of the remaining H atoms were placed in their calculated positions and then refined using the riding model with Atom—H lengths of 0.93Å (CH), 0.96Å (CH_2_) or 0.97Å (CH_3_). Isotropic displacement parameters for these atoms were set to 1.18–1.21 (CH, CH_2_) or 1.49–1.50 (CH_3_) times *U*_eq_ of the parent atom.

### Crystal data

**Table:**

C_29_H_33_ClN_2_O_2_·H_2_O	*F*(000) = 2112
*M**_r_* = 495.04	*D*_x_ = 1.251 Mg m^−^^3^
Orthorhombic, *P**b**c**a*	Mo *K*α radiation, λ = 0.71073 Å
Hall symbol: -P 2ac 2ab	Cell parameters from 15584 reflections
*a* = 16.7869 (4) Å	θ = 3.1–32.3°
*b* = 15.1506 (6) Å	µ = 0.18 mm^−^^1^
*c* = 20.6617 (6) Å	*T* = 170 K
*V* = 5254.9 (3) Å^3^	Chunk, colorless
*Z* = 8	0.45 × 0.30 × 0.20 mm

### Data collection

**Table:**

Oxford Diffraction Xcalibur Eos Gemini diffractometer	6247 independent reflections
Radiation source: Enhance (Mo) X-ray Source	5231 reflections with *I* > 2σ(*I*)
graphite	*R*_int_ = 0.033
Detector resolution: 16.1500 pixels mm^-1^	θ_max_ = 27.9°, θ_min_ = 3.1°
ω scans	*h* = −22→22
Absorption correction: multi-scan (*CrysAlis RED*; Oxford Diffraction, 2010)	*k* = −19→19
*T*_min_ = 0.989, *T*_max_ = 1.000	*l* = −27→27
48373 measured reflections	

### Refinement

**Table:**

Refinement on *F*^2^	Primary atom site location: structure-invariant direct methods
Least-squares matrix: full	Secondary atom site location: difference Fourier map
*R*[*F*^2^ > 2σ(*F*^2^)] = 0.044	Hydrogen site location: difference Fourier map
*wR*(*F*^2^) = 0.107	H atoms treated by a mixture of independent and constrained refinement
*S* = 1.04	*w* = 1/[σ^2^(*F*_o_^2^) + (0.0415*P*)^2^ + 2.2656*P*] where *P* = (*F*_o_^2^ + 2*F*_c_^2^)/3
6247 reflections	(Δ/σ)_max_ = 0.001
330 parameters	Δρ_max_ = 0.27 e Å^−^^3^
4 restraints	Δρ_min_ = −0.29 e Å^−^^3^

### Special details

**Table:**

Geometry. All esds (except the esd in the dihedral angle between two l.s. planes) are estimated using the full covariance matrix. The cell esds are taken into account individually in the estimation of esds in distances, angles and torsion angles; correlations between esds in cell parameters are only used when they are defined by crystal symmetry. An approximate (isotropic) treatment of cell esds is used for estimating esds involving l.s. planes.
Refinement. Refinement of F^2^ against ALL reflections. The weighted R-factor wR and goodness of fit S are based on F^2^, conventional R-factors R are based on F, with F set to zero for negative F^2^. The threshold expression of F^2^ > 2sigma(F^2^) is used only for calculating R-factors(gt) etc. and is not relevant to the choice of reflections for refinement. R-factors based on F^2^ are statistically about twice as large as those based on F, and R- factors based on ALL data will be even larger.

### Fractional atomic coordinates and isotropic or equivalent isotropic displacement parameters (Å^2^)

**Table:**

	*x*	*y*	*z*	*U*_iso_*/*U*_eq_	
Cl1	1.17722 (2)	0.58038 (4)	0.26920 (2)	0.06055 (15)	
N1	0.47942 (7)	0.28878 (9)	0.58732 (6)	0.0379 (3)	
N2	0.74378 (6)	0.48771 (7)	0.48602 (5)	0.0261 (2)	
O1	0.56971 (7)	0.30669 (7)	0.50943 (5)	0.0394 (3)	
O2	0.85002 (6)	0.68017 (7)	0.44131 (5)	0.0367 (2)	
H2O	0.8828 (11)	0.7037 (13)	0.4658 (9)	0.059 (6)*	
O1W	0.75307 (10)	0.33121 (10)	0.40445 (7)	0.0636 (4)	
H1W	0.7543 (17)	0.3701 (14)	0.4371 (11)	0.101 (9)*	
H2W	0.7248 (16)	0.2875 (14)	0.4215 (13)	0.114 (11)*	
C1	1.09073 (8)	0.58308 (12)	0.31591 (7)	0.0390 (4)	
C2	1.06820 (9)	0.66035 (11)	0.34473 (8)	0.0435 (4)	
H2	1.0998	0.7104	0.3408	0.052*	
C3	0.99766 (9)	0.66366 (10)	0.37999 (8)	0.0369 (3)	
H3	0.9822	0.7165	0.3992	0.044*	
C4	0.95013 (8)	0.58960 (9)	0.38694 (6)	0.0288 (3)	
C5	0.97593 (9)	0.51173 (11)	0.35815 (8)	0.0417 (4)	
H5	0.9455	0.4609	0.3630	0.050*	
C6	1.04572 (10)	0.50782 (12)	0.32247 (8)	0.0454 (4)	
H6	1.0618	0.4552	0.3033	0.054*	
C7	0.87288 (8)	0.59170 (9)	0.42628 (6)	0.0269 (3)	
C8	0.80218 (8)	0.55421 (10)	0.38837 (6)	0.0303 (3)	
H8A	0.7906	0.5926	0.3520	0.036*	
H8B	0.8163	0.4966	0.3714	0.036*	
C9	0.72810 (8)	0.54558 (10)	0.43032 (6)	0.0305 (3)	
H9A	0.7120	0.6035	0.4456	0.037*	
H9B	0.6848	0.5214	0.4048	0.037*	
C10	0.80641 (9)	0.52790 (11)	0.52601 (6)	0.0335 (3)	
H10A	0.8157	0.4914	0.5638	0.040*	
H10B	0.7888	0.5854	0.5408	0.040*	
C11	0.88357 (8)	0.53792 (10)	0.48847 (6)	0.0326 (3)	
H11A	0.9038	0.4798	0.4775	0.039*	
H11B	0.9228	0.5667	0.5157	0.039*	
C12	0.67039 (8)	0.47411 (9)	0.52392 (7)	0.0298 (3)	
H12A	0.6268	0.4596	0.4950	0.036*	
H12B	0.6568	0.5282	0.5465	0.036*	
C13	0.68173 (8)	0.39967 (9)	0.57288 (6)	0.0287 (3)	
H13A	0.7225	0.4176	0.6035	0.034*	
H13B	0.7017	0.3483	0.5500	0.034*	
C14	0.60632 (7)	0.37140 (8)	0.61187 (6)	0.0246 (3)	
C15	0.57026 (8)	0.45613 (8)	0.63995 (6)	0.0256 (3)	
C16	0.49950 (9)	0.49212 (10)	0.61733 (7)	0.0354 (3)	
H16	0.4701	0.4617	0.5863	0.042*	
C17	0.47176 (10)	0.57271 (11)	0.64016 (8)	0.0428 (4)	
H17	0.4241	0.5956	0.6246	0.051*	
C18	0.51458 (10)	0.61866 (10)	0.68566 (8)	0.0403 (4)	
H18	0.4953	0.6718	0.7018	0.048*	
C19	0.58649 (9)	0.58545 (10)	0.70730 (8)	0.0371 (3)	
H19	0.6165	0.6172	0.7371	0.044*	
C20	0.61395 (8)	0.50510 (9)	0.68474 (7)	0.0307 (3)	
H20	0.6624	0.4833	0.6997	0.037*	
C21	0.63539 (8)	0.30328 (9)	0.66256 (6)	0.0260 (3)	
C22	0.67485 (10)	0.22848 (10)	0.63995 (7)	0.0363 (3)	
H22	0.6805	0.2201	0.5956	0.044*	
C23	0.70578 (11)	0.16645 (11)	0.68201 (8)	0.0437 (4)	
H23	0.7320	0.1171	0.6658	0.052*	
C24	0.69791 (10)	0.17757 (10)	0.74803 (8)	0.0394 (3)	
H24	0.7197	0.1367	0.7765	0.047*	
C25	0.65740 (9)	0.24984 (10)	0.77106 (7)	0.0345 (3)	
H25	0.6511	0.2572	0.8155	0.041*	
C26	0.62582 (8)	0.31198 (9)	0.72898 (6)	0.0300 (3)	
H26	0.5979	0.3600	0.7454	0.036*	
C27	0.54922 (8)	0.32055 (9)	0.56588 (6)	0.0289 (3)	
C28	0.43120 (11)	0.23911 (14)	0.54098 (10)	0.0598 (5)	
H28A	0.4215	0.2748	0.5034	0.090*	
H28B	0.3813	0.2234	0.5606	0.090*	
H28C	0.4591	0.1865	0.5285	0.090*	
C29	0.44931 (10)	0.29174 (13)	0.65343 (9)	0.0502 (4)	
H29A	0.4573	0.2354	0.6737	0.075*	
H29B	0.3935	0.3053	0.6528	0.075*	
H29C	0.4773	0.3364	0.6773	0.075*	

### Atomic displacement parameters (Å^2^)

**Table:**

	*U*^11^	*U*^22^	*U*^33^	*U*^12^	*U*^13^	*U*^23^
Cl1	0.0316 (2)	0.0921 (4)	0.0580 (3)	0.0086 (2)	0.01224 (18)	0.0166 (2)
N1	0.0325 (6)	0.0378 (7)	0.0433 (7)	−0.0056 (5)	0.0020 (5)	−0.0085 (6)
N2	0.0259 (5)	0.0302 (6)	0.0222 (5)	−0.0031 (4)	0.0004 (4)	0.0026 (4)
O1	0.0500 (6)	0.0377 (6)	0.0304 (5)	−0.0004 (5)	0.0027 (5)	−0.0083 (4)
O2	0.0356 (5)	0.0290 (5)	0.0454 (6)	−0.0024 (4)	0.0008 (5)	−0.0055 (5)
O1W	0.0782 (10)	0.0581 (9)	0.0545 (8)	−0.0109 (8)	0.0067 (7)	−0.0136 (7)
C1	0.0258 (7)	0.0588 (10)	0.0326 (7)	0.0022 (7)	0.0012 (6)	0.0100 (7)
C2	0.0328 (8)	0.0425 (9)	0.0551 (10)	−0.0067 (7)	0.0044 (7)	0.0118 (7)
C3	0.0348 (7)	0.0325 (8)	0.0433 (8)	−0.0040 (6)	0.0021 (6)	0.0036 (6)
C4	0.0292 (6)	0.0325 (7)	0.0247 (6)	−0.0029 (5)	−0.0019 (5)	0.0021 (5)
C5	0.0376 (8)	0.0384 (8)	0.0491 (9)	−0.0082 (7)	0.0070 (7)	−0.0096 (7)
C6	0.0379 (8)	0.0505 (10)	0.0478 (9)	0.0008 (7)	0.0060 (7)	−0.0130 (8)
C7	0.0281 (6)	0.0262 (6)	0.0264 (6)	−0.0038 (5)	0.0007 (5)	−0.0002 (5)
C8	0.0325 (7)	0.0359 (7)	0.0226 (6)	−0.0066 (6)	−0.0026 (5)	0.0036 (5)
C9	0.0287 (7)	0.0345 (7)	0.0282 (6)	−0.0031 (6)	−0.0035 (5)	0.0058 (6)
C10	0.0352 (7)	0.0433 (8)	0.0221 (6)	−0.0091 (6)	−0.0025 (5)	0.0014 (6)
C11	0.0293 (7)	0.0406 (8)	0.0280 (6)	−0.0061 (6)	−0.0050 (5)	0.0047 (6)
C12	0.0272 (6)	0.0314 (7)	0.0309 (7)	0.0015 (5)	0.0046 (5)	0.0038 (5)
C13	0.0269 (6)	0.0313 (7)	0.0280 (6)	0.0026 (5)	0.0060 (5)	0.0035 (5)
C14	0.0252 (6)	0.0237 (6)	0.0249 (6)	0.0019 (5)	0.0032 (5)	0.0000 (5)
C15	0.0260 (6)	0.0238 (6)	0.0269 (6)	0.0008 (5)	0.0048 (5)	0.0014 (5)
C16	0.0337 (7)	0.0324 (7)	0.0400 (8)	0.0046 (6)	−0.0053 (6)	−0.0037 (6)
C17	0.0365 (8)	0.0348 (8)	0.0571 (10)	0.0116 (7)	−0.0044 (7)	−0.0015 (7)
C18	0.0440 (8)	0.0253 (7)	0.0515 (9)	0.0068 (6)	0.0079 (7)	−0.0037 (6)
C19	0.0392 (8)	0.0293 (7)	0.0427 (8)	−0.0036 (6)	0.0002 (6)	−0.0069 (6)
C20	0.0275 (6)	0.0283 (7)	0.0363 (7)	0.0003 (5)	0.0008 (5)	−0.0009 (6)
C21	0.0261 (6)	0.0241 (6)	0.0277 (6)	−0.0002 (5)	0.0018 (5)	0.0005 (5)
C22	0.0482 (8)	0.0329 (8)	0.0280 (7)	0.0101 (6)	0.0012 (6)	−0.0027 (6)
C23	0.0559 (10)	0.0341 (8)	0.0411 (8)	0.0172 (7)	−0.0005 (7)	−0.0005 (7)
C24	0.0463 (8)	0.0352 (8)	0.0369 (7)	0.0051 (7)	−0.0063 (7)	0.0078 (6)
C25	0.0403 (8)	0.0370 (8)	0.0263 (6)	−0.0042 (6)	0.0009 (6)	0.0034 (6)
C26	0.0335 (7)	0.0266 (7)	0.0299 (7)	−0.0005 (6)	0.0066 (5)	−0.0011 (5)
C27	0.0326 (7)	0.0238 (6)	0.0302 (7)	0.0043 (5)	−0.0001 (5)	−0.0019 (5)
C28	0.0468 (10)	0.0601 (12)	0.0723 (12)	−0.0139 (9)	−0.0054 (9)	−0.0236 (10)
C29	0.0404 (9)	0.0603 (11)	0.0500 (10)	−0.0135 (8)	0.0127 (7)	−0.0020 (8)

### Geometric parameters (Å, °)

**Table:**

Cl1—C1	1.7440 (15)	C12—H12A	0.9700
N1—C27	1.3419 (18)	C12—H12B	0.9700
N1—C29	1.457 (2)	C13—C14	1.5606 (17)
N1—C28	1.462 (2)	C13—H13A	0.9700
N2—C10	1.4693 (17)	C13—H13B	0.9700
N2—C9	1.4705 (16)	C14—C15	1.5332 (17)
N2—C12	1.4743 (16)	C14—C21	1.5493 (18)
O1—C27	1.2340 (16)	C14—C27	1.5542 (18)
O2—C7	1.4284 (17)	C15—C16	1.3880 (19)
O2—H2O	0.827 (15)	C15—C20	1.3946 (19)
O1W—H1W	0.896 (16)	C16—C17	1.389 (2)
O1W—H2W	0.888 (16)	C16—H16	0.9300
C1—C2	1.367 (2)	C17—C18	1.373 (2)
C1—C6	1.375 (2)	C17—H17	0.9300
C2—C3	1.391 (2)	C18—C19	1.382 (2)
C2—H2	0.9300	C18—H18	0.9300
C3—C4	1.384 (2)	C19—C20	1.383 (2)
C3—H3	0.9300	C19—H19	0.9300
C4—C5	1.390 (2)	C20—H20	0.9300
C4—C7	1.5307 (18)	C21—C26	1.3879 (18)
C5—C6	1.386 (2)	C21—C22	1.3934 (19)
C5—H5	0.9300	C22—C23	1.381 (2)
C6—H6	0.9300	C22—H22	0.9300
C7—C8	1.5312 (18)	C23—C24	1.381 (2)
C7—C11	1.5321 (18)	C23—H23	0.9300
C8—C9	1.5214 (19)	C24—C25	1.374 (2)
C8—H8A	0.9700	C24—H24	0.9300
C8—H8B	0.9700	C25—C26	1.387 (2)
C9—H9A	0.9700	C25—H25	0.9300
C9—H9B	0.9700	C26—H26	0.9300
C10—C11	1.5174 (19)	C28—H28A	0.9600
C10—H10A	0.9700	C28—H28B	0.9600
C10—H10B	0.9700	C28—H28C	0.9600
C11—H11A	0.9700	C29—H29A	0.9600
C11—H11B	0.9700	C29—H29B	0.9600
C12—C13	1.5269 (18)	C29—H29C	0.9600
			
C27—N1—C29	126.97 (13)	C12—C13—H13A	108.2
C27—N1—C28	116.86 (13)	C14—C13—H13A	108.2
C29—N1—C28	115.94 (14)	C12—C13—H13B	108.2
C10—N2—C9	108.73 (11)	C14—C13—H13B	108.2
C10—N2—C12	110.93 (10)	H13A—C13—H13B	107.4
C9—N2—C12	110.46 (10)	C15—C14—C21	115.23 (10)
C7—O2—H2O	111.0 (15)	C15—C14—C27	113.76 (10)
H1W—O1W—H2W	101.9 (19)	C21—C14—C27	106.10 (10)
C2—C1—C6	121.03 (14)	C15—C14—C13	106.61 (10)
C2—C1—Cl1	119.44 (13)	C21—C14—C13	106.04 (10)
C6—C1—Cl1	119.52 (13)	C27—C14—C13	108.71 (10)
C1—C2—C3	119.64 (15)	C16—C15—C20	117.68 (12)
C1—C2—H2	120.2	C16—C15—C14	122.64 (12)
C3—C2—H2	120.2	C20—C15—C14	119.27 (11)
C4—C3—C2	121.05 (15)	C15—C16—C17	121.18 (14)
C4—C3—H3	119.5	C15—C16—H16	119.4
C2—C3—H3	119.5	C17—C16—H16	119.4
C3—C4—C5	117.63 (13)	C18—C17—C16	120.17 (14)
C3—C4—C7	121.77 (13)	C18—C17—H17	119.9
C5—C4—C7	120.58 (12)	C16—C17—H17	119.9
C6—C5—C4	121.82 (15)	C17—C18—C19	119.61 (14)
C6—C5—H5	119.1	C17—C18—H18	120.2
C4—C5—H5	119.1	C19—C18—H18	120.2
C1—C6—C5	118.80 (16)	C18—C19—C20	120.19 (14)
C1—C6—H6	120.6	C18—C19—H19	119.9
C5—C6—H6	120.6	C20—C19—H19	119.9
O2—C7—C4	111.26 (11)	C19—C20—C15	121.11 (13)
O2—C7—C8	104.54 (11)	C19—C20—H20	119.4
C4—C7—C8	112.17 (11)	C15—C20—H20	119.4
O2—C7—C11	110.37 (11)	C26—C21—C22	117.64 (12)
C4—C7—C11	109.57 (11)	C26—C21—C14	124.66 (12)
C8—C7—C11	108.81 (11)	C22—C21—C14	117.70 (11)
C9—C8—C7	111.97 (11)	C23—C22—C21	121.41 (13)
C9—C8—H8A	109.2	C23—C22—H22	119.3
C7—C8—H8A	109.2	C21—C22—H22	119.3
C9—C8—H8B	109.2	C24—C23—C22	120.18 (14)
C7—C8—H8B	109.2	C24—C23—H23	119.9
H8A—C8—H8B	107.9	C22—C23—H23	119.9
N2—C9—C8	110.54 (11)	C25—C24—C23	119.12 (14)
N2—C9—H9A	109.5	C25—C24—H24	120.4
C8—C9—H9A	109.5	C23—C24—H24	120.4
N2—C9—H9B	109.5	C24—C25—C26	120.86 (13)
C8—C9—H9B	109.5	C24—C25—H25	119.6
H9A—C9—H9B	108.1	C26—C25—H25	119.6
N2—C10—C11	111.40 (11)	C25—C26—C21	120.74 (13)
N2—C10—H10A	109.3	C25—C26—H26	119.6
C11—C10—H10A	109.3	C21—C26—H26	119.6
N2—C10—H10B	109.3	O1—C27—N1	119.63 (13)
C11—C10—H10B	109.3	O1—C27—C14	119.36 (12)
H10A—C10—H10B	108.0	N1—C27—C14	120.96 (12)
C10—C11—C7	112.46 (11)	N1—C28—H28A	109.5
C10—C11—H11A	109.1	N1—C28—H28B	109.5
C7—C11—H11A	109.1	H28A—C28—H28B	109.5
C10—C11—H11B	109.1	N1—C28—H28C	109.5
C7—C11—H11B	109.1	H28A—C28—H28C	109.5
H11A—C11—H11B	107.8	H28B—C28—H28C	109.5
N2—C12—C13	110.54 (11)	N1—C29—H29A	109.5
N2—C12—H12A	109.5	N1—C29—H29B	109.5
C13—C12—H12A	109.5	H29A—C29—H29B	109.5
N2—C12—H12B	109.5	N1—C29—H29C	109.5
C13—C12—H12B	109.5	H29A—C29—H29C	109.5
H12A—C12—H12B	108.1	H29B—C29—H29C	109.5
C12—C13—C14	116.33 (11)		
			
C6—C1—C2—C3	−1.6 (2)	C13—C14—C15—C16	108.68 (14)
Cl1—C1—C2—C3	177.56 (12)	C21—C14—C15—C20	53.60 (16)
C1—C2—C3—C4	0.6 (2)	C27—C14—C15—C20	176.46 (12)
C2—C3—C4—C5	0.8 (2)	C13—C14—C15—C20	−63.74 (15)
C2—C3—C4—C7	179.21 (13)	C20—C15—C16—C17	−2.2 (2)
C3—C4—C5—C6	−1.3 (2)	C14—C15—C16—C17	−174.70 (14)
C7—C4—C5—C6	−179.78 (14)	C15—C16—C17—C18	0.3 (3)
C2—C1—C6—C5	1.0 (3)	C16—C17—C18—C19	1.8 (3)
Cl1—C1—C6—C5	−178.10 (13)	C17—C18—C19—C20	−2.0 (2)
C4—C5—C6—C1	0.5 (3)	C18—C19—C20—C15	0.1 (2)
C3—C4—C7—O2	12.31 (18)	C16—C15—C20—C19	1.9 (2)
C5—C4—C7—O2	−169.32 (13)	C14—C15—C20—C19	174.73 (13)
C3—C4—C7—C8	129.02 (14)	C15—C14—C21—C26	4.52 (18)
C5—C4—C7—C8	−52.61 (17)	C27—C14—C21—C26	−122.32 (14)
C3—C4—C7—C11	−110.02 (15)	C13—C14—C21—C26	122.19 (14)
C5—C4—C7—C11	68.35 (16)	C15—C14—C21—C22	−174.61 (12)
O2—C7—C8—C9	−66.58 (14)	C27—C14—C21—C22	58.54 (15)
C4—C7—C8—C9	172.74 (11)	C13—C14—C21—C22	−56.95 (15)
C11—C7—C8—C9	51.34 (15)	C26—C21—C22—C23	−2.1 (2)
C10—N2—C9—C8	62.26 (14)	C14—C21—C22—C23	177.10 (15)
C12—N2—C9—C8	−175.80 (11)	C21—C22—C23—C24	0.1 (3)
C7—C8—C9—N2	−58.86 (15)	C22—C23—C24—C25	1.5 (3)
C9—N2—C10—C11	−61.25 (15)	C23—C24—C25—C26	−1.1 (2)
C12—N2—C10—C11	177.10 (12)	C24—C25—C26—C21	−0.9 (2)
N2—C10—C11—C7	56.54 (17)	C22—C21—C26—C25	2.5 (2)
O2—C7—C11—C10	64.01 (15)	C14—C21—C26—C25	−176.64 (13)
C4—C7—C11—C10	−173.13 (12)	C29—N1—C27—O1	173.93 (16)
C8—C7—C11—C10	−50.16 (16)	C28—N1—C27—O1	−0.3 (2)
C10—N2—C12—C13	−71.26 (15)	C29—N1—C27—C14	−3.6 (2)
C9—N2—C12—C13	168.11 (11)	C28—N1—C27—C14	−177.83 (14)
N2—C12—C13—C14	−174.15 (11)	C15—C14—C27—O1	121.16 (13)
C12—C13—C14—C15	−50.72 (15)	C21—C14—C27—O1	−111.12 (13)
C12—C13—C14—C21	−173.99 (11)	C13—C14—C27—O1	2.55 (17)
C12—C13—C14—C27	72.30 (15)	C15—C14—C27—N1	−61.33 (16)
C21—C14—C15—C16	−133.97 (13)	C21—C14—C27—N1	66.39 (15)
C27—C14—C15—C16	−11.12 (18)	C13—C14—C27—N1	−179.94 (12)

### Hydrogen-bond geometry (Å, °)

**Table:**

*D*—H···*A*	*D*—H	H···*A*	*D*···*A*	*D*—H···*A*
O1W—H2W···O2^i^	0.89 (2)	2.10 (2)	2.9684 (19)	168 (3)
O1W—H1W···N2	0.90 (2)	2.06 (2)	2.9132 (18)	160 (2)
O2—H2O···O1^ii^	0.83 (2)	1.97 (2)	2.7333 (15)	153 (2)
C28—H28B···O1W^iii^	0.96	2.42	3.369 (3)	171.

Symmetry codes: (i) −*x*+3/2, *y*−1/2, *z*; (ii) −*x*+3/2, *y*+1/2, *z*; (iii) *x*−1/2, −*y*+1/2, −*z*+1.
